# Quantifying Susceptibility of CD4^+^ Stem Memory T-Cells to Infection by Laboratory Adapted and Clinical HIV-1 Strains

**DOI:** 10.3390/v6020709

**Published:** 2014-02-10

**Authors:** Jacqueline K. Flynn, Geza Paukovics, Kieran Cashin, Katharina Borm, Anne Ellett, Michael Roche, Martin R. Jakobsen, Melissa J. Churchill, Paul R. Gorry

**Affiliations:** 1 Center for Biomedical Research, Burnet Institute, Melbourne, Victoria 3004, Australia; E-Mails: jflynn@burnet.edu.au (J.K.F.); Kieran@burnet.edu.au (K.C.); katharinab@burnet.edu.au (K.B.); amellett@burnet.edu.au (A.E.); mroche@burnet.edu.au (M.R.); churchil@burnet.edu.au (M.J.C.); 2 Department of Infectious Diseases, Monash University, Melbourne, Victoria 3004, Australia; 3 Alfred Medical Research and Education Precinct and Burnet Institute Flow Cytometry Core Facility, Melbourne, Victoria 3004, Australia; E-Mail: paukovic@burnet.edu.au; 4 Department of Microbiology and Immunology, University of Melbourne, Parkville, Victoria 3010, Australia; 5 Department of Microbiology, La Trobe University, Melbourne, Victoria 3086, Australia; 6 Department of Biomedicine, Aarhus University, Aarhus 237551, Denmark; E-Mail: mrj@immunology.au.dk; 7 Department of Medicine, Monash University, Melbourne, Victoria 3004, Australia; 8 Department of Microbiology, Monash University, Melbourne, Victoria 3010, Australia

**Keywords:** HIV-1, stem memory T cells, CD4^+^ T cells, T cell subsets, envelope, viral reservoir

## Abstract

CD4^+^ T cells are principal targets for human immunodeficiency virus type 1 (HIV-1) infection. CD4^+^ T cell subsets are heterogeneous cell populations, divided by functional and phenotypic differences into naïve and memory T cells. The memory CD4^+^ T cells are further segregated into central, effector and transitional memory cell subsets by functional, phenotypic and homeostatic characteristics. Defining the distribution of HIV-1 infection in different T cell subsets is important, as this can play a role in determining the size and composition of the viral reservoir. Both central memory and transitional memory CD4^+^ T cells have been described as long-lived viral reservoirs for HIV. Recently, the newly described stem memory T cell subset has also been implicated as a long-lived HIV reservoir. Using green fluorescent protein (GFP) reporter strains of HIV-1 and multi parameter flow cytometry, we developed an assay to simultaneously quantify the susceptibility of stem memory (TSCM), central memory, effector memory, transitional memory and naïve CD4^+^ T cell subsets, to HIV-1 infection *in vitro*. We show that TSCM are susceptible to infection with laboratory adapted and clinical HIV-1 strains. Our system facilitates the quantitation of HIV-1 infection in alternative T cell subsets by CCR5- and CXCR4-using viruses across different HIV-1 subtypes, and will be useful for studies of HIV-1 pathogenesis and viral reservoirs.

## 1. Introduction

Memory T cells play an important part of the adaptive immune response to infection [1,2,3]. Upon antigen encounter, naïve T cells undergo proliferation and differentiation into different memory T cell subsets which culminate into terminally differentiated effector T cells [4,5]. During the process of maturation, T cells progressively acquire effector functions but also lose the capacity for self-renewal and survival [1]. A small proportion of memory T cells survives the contraction phase and become long-lived memory T cells which have the ability to rapidly acquire effector functions upon reinfection [3,4].

Memory CD4^+^ T cells have conventionally been divided into central memory (CM) and effector memory (EM) based on their surface receptor expression (CCR7, CD62L) and the level and type of cytokine secretion (IFN-γ, IL-2, IL-4) [6,7]. CM CD4^+^ T cells are relatively long-lived memory cells which are able to undergo differentiation into a shorter lived EM T cells upon antigen stimulation [1,5,7] and, although to a lesser extent, in response homeostatic cytokines (for example IL-7 and IL-15) [8,9]. Transitional memory (TM) CD4^+^ T cells have functional and transcriptional characteristics which are in between those of CM and EM T cells [10] and can be distinguished through the additional use of CD27 surface receptor expression [8]. More recently a novel T cell subset, stem memory T cells (TSCM) has been detected in both CD4^+^ and CD8^+^ T cell populations in humans and nonhuman primates [11,12].

This novel T cell subset comprises ^~^2%–3% of the CD4^+^ and CD8^+^ T cell population in healthy individuals, and exhibits a gene profile between naïve and CM T cells [11,12]. These cells constitute a small proportion of the memory T cell subset and display stem cell-like properties, representing the earliest and longest lasting developmental stage of memory T cells [12]. TSCM cells display common surface markers characteristic of naïve T cells (CD45RA^+^, CD45RO^−^, CCR7^+^, CD27^+^), but can be distinguished from naïve T cells by high expression of CD95 and CD122 (IL-2Rβ) on their cellular surface, markers which are also expressed by memory T cell subsets [11]. TSCM are antigen‑experienced and upon TCR stimulation, exhibit effector activity and are able to differentiate into CM and EM subsets. They have the ability to self-renew in the presence of IL-15 homeostatic signals, and are able to survive for longer periods than CM or EM populations [12].

Previous studies have demonstrated the importance of memory T cells in immune responses to viral infections [2,13]. In HIV-1, CD4^+^ T cells are a key target of infection, where depletion of these cells results in deterioration of the immune system and progression to AIDS [14,15]. Importantly, both CM and TM CD4^+^ T cell subsets have been demonstrated as major HIV-1 cellular reservoirs where the maintenance of these reservoirs is associated with T cell survival and homeostatic proliferation (antigen-driven and IL-7-mediated, respectively) [8]. Additionally, the TSCM subset has been demonstrated to support long-lived T cell memory, with the potential to be a cellular reservoir of HIV‑1 [12,16,17].

Preliminary studies have demonstrated promising results for TSCM with a particular interest in the role of these cells in cancer and HIV-1. In mouse models, TSCM have exhibited increased anti-tumor activity and are being considered for adoptive T cell therapies for cancer patients [1,12,18]. Importantly for HIV-1 research, recent studies [17] have demonstrated CD4^+^ TSCM to contain high per-cell levels of HIV-1 DNA and contribute to the total CD4^+^ T cell reservoir. Thus, long-lived TSCM have the potential to promote HIV-1 viral persistence [17,19].

The cellular tropism of HIV-1 can influence the size and composition of the viral reservoir, with particular CD4^+^ T cell subsets described as cellular reservoirs for HIV-1 [8,20]. How the cellular tropism of HIV-1 for different T cell subtypes alters during disease pathogenesis is largely unknown. Thus, the development of an assay system which has the ability to detect changes in HIV-1 tropism for T cells during disease pathogenesis, and also characterizes changes in tropism between CCR5- and CXCR4-using viruses is important for the design of new therapeutic targets and for characterizing the cellular reservoir of HIV-1.

## 2. Results and Discussion

### 2.1. Development and Validation of a T Cell Assay for the Detection of Infected TSCM

We previously developed an assay to quantify HIV-1 infection in non-TSCM CD4^+^ T cell subsets *in vitro* [21]. This assay detected and quantified HIV-1 infection in CM, TM, EM, naïve and effector memory RA (EMRA) CD4^+^ T cells [21]. In this previous system the CD4^+^ T cells were activated with anti-CD3 and anti-CD28 (5 μg/mL) prior to infection with Env-pseudotyped GFP reporter viruses. The CD4^+^ T cells were cultured in media supplemented with IL-2 (20 U/mL) at all stages of the experiment (described in [21]). Since the recent description of TSCM cells, we have developed a new assay system which incorporates quantitation of HIV-1 infection in the TSCM subset.

**Figure 1:**
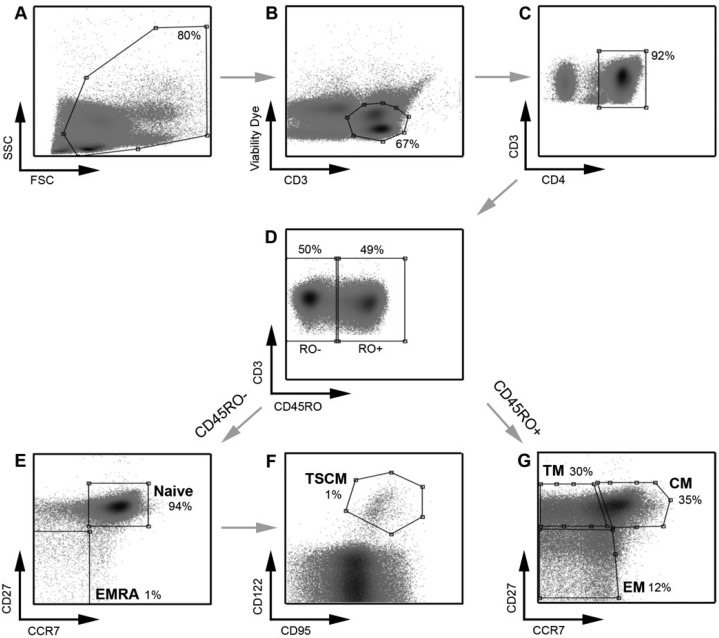
Strategy for identifying CD4^+^ T cell subsets. PBMC were stained with a panel of flow cytometry antibodies for the detection of CD4^+^ T cell subsets. PBMC were first gated on (**A**) FSC *vs*. SSC and then (**B**) viability of CD3^+^ T cells (CD3 *vs*. Viability dye). (**C**) Viable CD3^+^ T cells were gated on CD4^+^ positivity to determine viable CD3^+^CD4^+^ T cells. (**D**) CD3^+^CD4^+^ T cells were gated on CD45RO expression (CD45RO *vs*. CD3) and divided into CD45RO positive and negative cells. Each population, CD45RO positive and negative, was then plotted CCR7 *vs*. CD27 to define each T cell subset. (**E**) CD45RO negative cells were divided into naïve (CD45RO^−^CCR7^+^CD27^+^) and effector (EMRA; CD45ROCCR7^+^CD27^−^) CD4^+^ T cells. (**F**) Cells in the naïve CD4^+^ T cell gate were further divided into TSCM cells using CD122 and CD95 (CD45RO^−^CCR7^+^CD27^+^CD122^+^CD95^+^) (**G**) CD45RO positive cells were divided into transitional memory (TM; CD45RO^+^CCR7^−^CD27^+^), central memory (CM; CD45RO^+^CCR7^+^CD27^+^) and effector memory (EM; CD45RO^+^CCR7^−^CD27^−^) CD4^+^ T cells. This gating strategy allowed EM to be distinguished from TM, similar to methods used by other investigators [8] and as we have described previously [21]. The use of an additional cellular marker, CD27, also enabled better enumeration of T cell subsets, as described previously [23,24]. TSCM were identified using methods similar to those described previously [11]. Percentages represent the percent of the parent population gated (for example, in plot **D**, CD45RO^−^ cells represent 50% of the CD3^+^CD4^+^ T cells gated in plot **C**).

TSCM cells are the least differentiated of the memory T cell populations [11]. They express many naïve markers and are relatively rare, comprising approximately 2%–4% of the total CD4^+^ T cells in the blood [11]. They can be differentiated from naïve T cells by the use of the memory marker CD95 and CD122 [11]. In developing the new assay, we first ensured detection of all CD4^+^ T cell subsets in uninfected CD4^+^ T cells from peripheral blood using a panel of cytometry antibodies (Table 1, Figure 1).

**Table 1:** Flow cytometry panel for the detection of CD4^+^ T cell subsets.

Cellular Marker	Fluorochrome
CD4	FITC
CD122	EF710 (PerCPCy5.5)
CCR7	AF-647
CD3	APCCy7
CD45RO	EF450 (Pacific Blue)
Viability Dye *	EF506 (Amcyan)
CD95	PE-CF594
CD27 ^	PE-Cy7
CCR5 ^#^	PE
CXCR4 ^#^	PECy5

* A fixable viability dye was chosen so the HIV-1 infected cells would also be fixed. ^ CD27 has been chosen over CD62L as expression of CD62L is not reliable on cryopreserved cells [22]. ^#^ CCR5 and CXCR4 antibodies used for HIV-1 infected samples.

**Figure 2:**
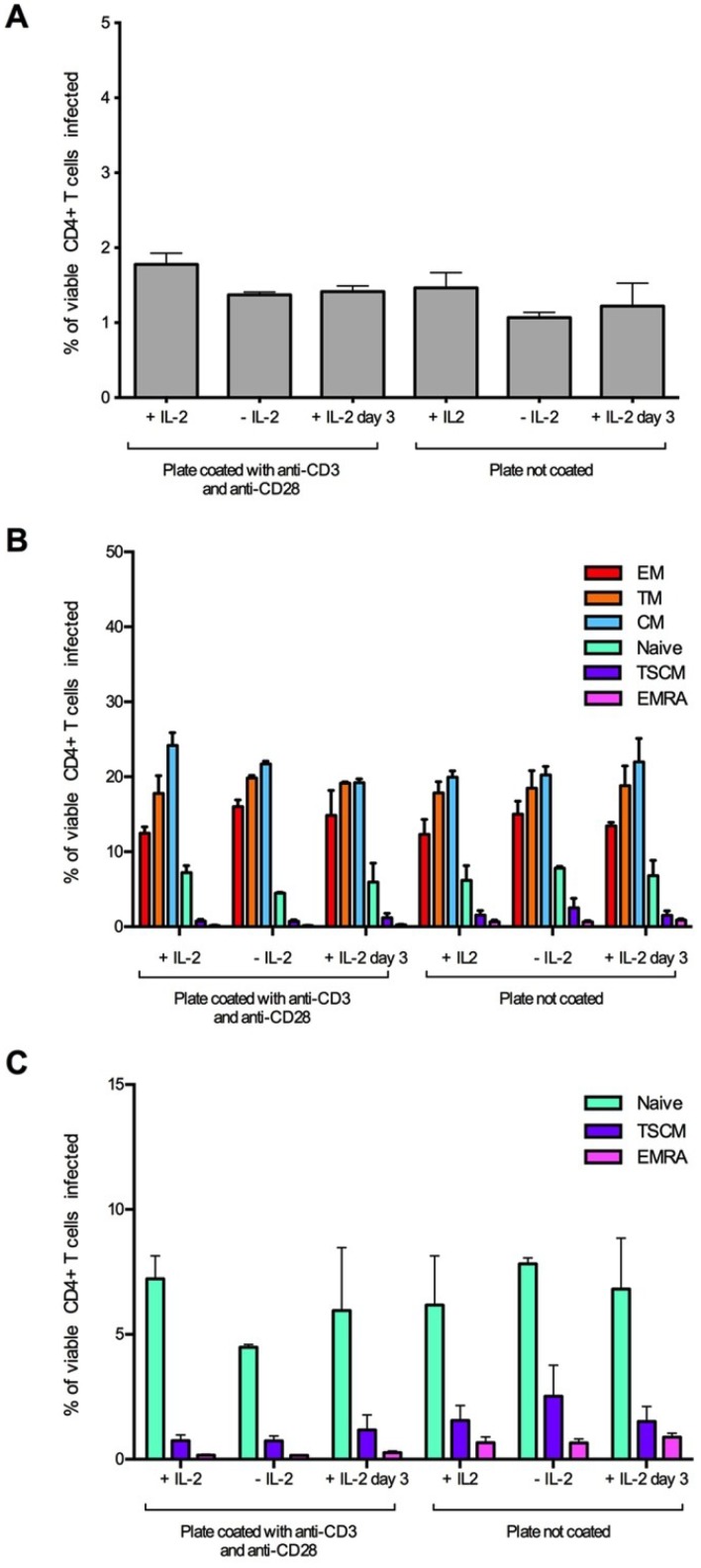
Establishment and optimization of assay conditions for the detection of HIV-1 infected TSCM cells. CD4^+^ T cells were isolated from two donors and infected with 3,000 IU of JR-CSF Env-pseudotyped GFP reporter virus. Cells were incubated for three days prior to infection on coated (anti-CD3 and anti-CD28, 5 μg/mL) or uncoated wells with and without the addition of IL-2 (20 U/mL) to the culture media. A third condition of the addition of IL-2 post-infection (day 3) was also tested. For all conditions, >1 million events were collected on a LSR Fortessa flow cytometer 3 days post infection. (**A**) There was little change in the level of infection between the assay conditions, although there was a slight reduction in infection in uncoated wells and in wells without IL-2. (**B**) There was little difference in the proportion of CD4^+^ T cell subset infection between the conditions; however, (**C**) there was a trend for an increased infection of TSCM from uncoated wells. Panel **C** represents the naïve, TSCM and EMRA T cell subset populations from panel **B** plotted on a smaller scale. Bar graphs represent the median and range.

Because TSCM share cellular markers with naïve T cells and are a relatively rare population, we proposed that the activation of CD4^+^ T cells prior to infection in our *in vitro* system may not accurately depict HIV-1 infection of this subset. Therefore, to examine the effect of stimulating CD4^+^ T cells prior to infection and the effect of the addition of IL-2 to the assay, we performed experiments with both stimulated (plates coated with anti-CD3 and anti-CD28) and unstimulated cells in the presence and absence of IL-2 (Figure 2). As expected, without anti-CD3 and anti-CD28 stimulation, and without the addition of IL-2, there was lower T cell infectivity (Figure 2A), however there was also a slight increase in the detection of HIV-1 infected TSCM (Figure 2C). There was little change in the proportion of CD4^+^ T cell subsets infected with or without prior stimulation or the addition of IL-2 (Figure 2B), thus we chose not to stimulate the CD4^+^ T cells in future experiments.

**Figure 3:**
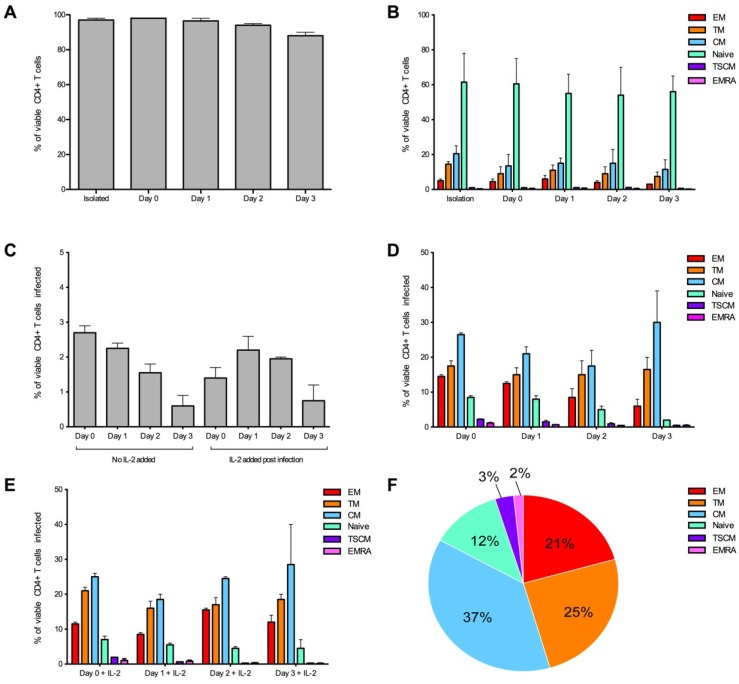
Optimization of the detection of infected CD4^+^ T cell subsets over time. CD4^+^ T cells from two donors were infected with 3,000 IU of JR-CSF Env-pseudotyped GFP reporter virus at day 0 (1 hour post isolation), day 1 (24 hours post isolation), day 2 (48 hours post isolation) and day 3 (72 hours post isolation). For all conditions, over 1 million events were collected on a LSR Fortessa 3 days post infection. (**A**) CD4^+^ T cells maintained a good level of viability (>95% viable) in experimental conditions day 0 and day 1. Viability was measured at the end of the experiment. (**B**) CD4^+^ T cell subset proportions were maintained in no virus control wells (assessed at the end of the experiment) and were similar to freshly isolated CD4^+^ T cells (isolation) at all conditions examined. (**C**) There was a trend for better consistency in the percentage of CD4^+^ T cells infected in wells without the addition of IL-2, and there was a tendency for a higher level of infection at earlier time points. There was little difference in proportion of T cell subsets infected when cells were (**D**) untreated or (**E**) treated with IL-2 post-infection. (**E**) There was a tendency for better consistency of subsets infected at earlier time points. (**F**) A representative pie chart displaying the proportion of CD4^+^ T cell subsets infected (using the condition of cells infected on day 0, without IL-2). Bar graphs represent the median and range.

We next performed time course experiments to determine the optimal time to infect the CD4^+^ T cells after isolation. We examined cell viability, infection levels and consistency of T cell subsets infected (Figure 3). These assays confirmed good viability, reproducible infection levels and the greatest consistency with infection of CD4^+^ T cell subsets when infection was on the same day of isolation (day 0) or 24 hours post isolation (day 1, Figure 3A,C–E). We also ensured T cell subset proportions of no virus control wells represented CD4^+^ T cell subset starting populations (Figure 3B). In all conditions the susceptibility of each T cell subset to infection remained consistent. CM cells were the most susceptible to infection by JR-CSF followed by TM, EM, naïve, TSCM and EMRA cells (Figure 3F). Due to the short assay duration (72 hours) and the choice of flow cytometry antibodies for detection of TSCM including CD122 (IL-2Rβ, Table 1), we chose not to add IL-2 to the assay at any stage and chose to infect the CD4^+^ T cells one hour post-isolation for the experimental protocol.

### 2.2. HIV-1 Infection in CD4^+^ T Cell Subsets by CCR5- and CXCR4-Using Viruses

**Figure 4:**
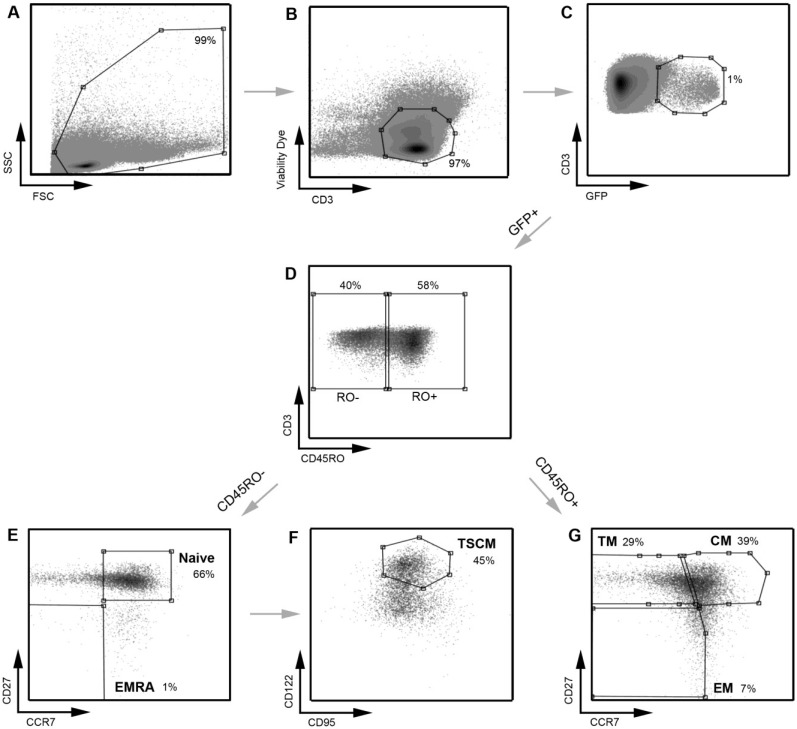
A representative example of the strategy used to define infected CD4^+^ T cell subsets. A similar gating strategy to that shown in Figure 1 was applied for the detection of infected CD4^+^ T cells. CD4^+^ T cells infected with a subtype C CCR5-using Env‑pseudotyped GFP reporter virus (258-E-5) were first gated on (**A**) FSC *vs*. SSC and then (**B**) viable CD3^+^ T cells (as shown in Figure 1). (**C**) HIV-1 infection was determined by CD3/GFP positivity and used to govern the distribution of infection within CD4^+^ T cell subsets. (**D**) CD3^+^CD4^+^GFP^+^ T cells were divided into CD45RO negative and positive cells. (**E**) CD45RO negative cells were divided into naïve (CD45RO^−^CCR7^+^CD27^+^) and effector (EMRA; CD45ROCCR7^+^CD27^−^) CD4^+^ T cells. (**F**) Using CD122 and CD95, CD4^+^ T cells in the naïve T cell gate were divided into TSCM cells (CD45RO^−^CCR7^+^CD27^+^CD122^+^CD95^+^) (**G**) CD45RO positive cells were divided into memory CD4^+^ T cell subsets; TM (CD45RO^+^CCR7^−^CD27^+^), CM (CD45RO^+^CCR7^+^CD27^+^) and EM (CD45RO^+^CCR7^−^CD27^−^). TSCM were identified using methods similar to those described previously [11]. Percentages represent the percent of the parent population gated.

We next confirmed that we could detect and measure HIV-1 infection of the CD4^+^ T cell subsets by both CCR5- and CXCR4-using viruses. The strategy for detecting HIV-1 infection in the different T cell subsets is illustrated in Figure 4. We used the well characterized JR-CSF (CCR5-using) and HXB2 (CXCR4-using) Envs to produce Env-pseudotyped GFP reporter viruses, to examine CD4^+^ T cell subset tropism and infectivity. JR-CSF and HXB2 are frequently used as controls in HIV-1 experiments. Both viruses are derived from patient isolates and are subtype B HIV-1. JR-CSF and HXB2 have been demonstrated to be T cell tropic [21,25,26].

Prior to infection we examined the level of CCR5 and CXCR4 expression on all CD4^+^ T cell subsets from five healthy donors. CD4 expression from the five donors was similar across all T cell subsets (as previously reported [21], and data not shown). CCR5 expression varied between T cell subsets with the highest expression on EM and TM cells (17 +/− 5% and 14 +/− 6%, respectively), a moderate expression on CM cells (9 +/− 5%), lower expression on TSCM (6 +/− 5%) and very low expression on naïve and EMRA (both 1 +/−1 %). CXCR4 expression was high on all T cell subsets (>70%) with the highest expression on naïve T cells (90 +/− 5%). The distribution of CD4, CCR5 and CXCR4 expression across the T cell subsets was similar to that reported previously for peripheral blood CD4^+^ T cell subsets [21,27,28,29].

**Figure 5:**
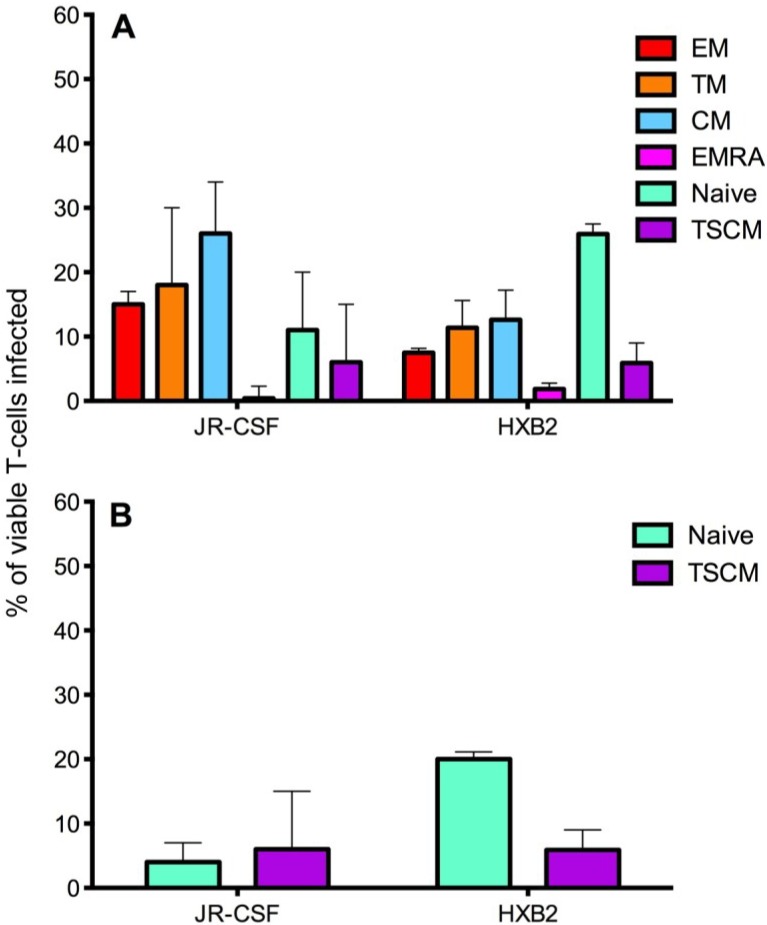
Infection of CD4^+^ T cell subsets by CCR5- and CXCR4-using viruses. CD4^+^ T cells were isolated from five donors and infected with JR-CSF (CCR5-using) or HXB2 (CXCR4-using) Env-pseudotyped GFP reporter viruses. Over 1 million events were collected on a LSR Fortessa flow cytometer 3 days post infection. (**A**) JR-CSF preferentially infected memory CD4^+^ T cells including CM, TM and EM. In contrast, HXB2 preferentially infected naïve T cells. Both viruses were able to infect TSCM. (**B**) Removal of JR-CSF infected TSCM (CD3^+^CD4^+^CD45RO^−^CD27^+^CCR7^+^CD122^+^CD95^+^) from the infected naïve T cell gate (CD3^+^CD4^+^CD45RO^−^CD27^+^CCR7^+^, as TSCM share the markers CD45RO^−^CD27^+^CCR7^+^ with naïve T cells) revealed a low level of infection of naïve T cells by JR-CSF, whereas HXB2 maintained a relatively high level of infection of naïve T cells. Percentages represent the percentage of naïve T cells infected by each virus, which are TSCM. Bar graphs represent the median and range.

CD4^+^ T cells from the five donors were infected with JR-CSF and HXB2 Env-pseudotyped GFP reporter viruses, and showed a similar level of overall infection ranging from 1 to 2% of total CD4^+^ T cells (JR-CSF mean 1.8 +/− 0.8% and HXB2 mean 1.0 +/− 0.3%). There was a difference in CD4^+^ T cell subset tropism between JR-CSF and HXB2, likely due to their difference in use of co-receptor for viral entry (Figure 5). The CCR5-using JR-CSF preferentially infected memory T cell subsets, in particular CM (mean 26.60 +/− 6.23%) and TM (mean 20.00 +/− 7.84%) with a lower level of infection for naïve T cells (mean 12.00 +/− 5.52%, Figure 5A). In contrast, the CXCR4-using HXB2 preferentially infected naïve T cells (mean 25.13 +/− 2.00%) with a lower level of infection in memory T cell subsets (CM mean 13.11 +/− 2.85%, TM mean 12.35 +/− 2.57%, Figure 5A). Both JR-CSF and HXB2 were able to infect a similar proportion of TSCM cells (JR-CSF mean 7.80 +/− 5.07%, HXB2 mean 6.32 +/− 1.65%, Figure 5A).

The preferential infection of memory T cell subsets by the CCR5-using JR-CSF could be facilitated by high expression of the co-receptor CCR5 on these subsets [21,27,28,29]. Similarly, the preferential infection of naïve T cells by the CXCR4-using HXB2 may have been facilitated by naïve T cells expressing higher levels of CXCR4 [27,28]. Both JR-CSF and HXB2 infected a similar proportion of TSCM cells, approximately 6%–7%; this level of infectivity by the CCR5-using JR-CSF could be contributed to by TSCM expressing a low level of CCR5 compared to other memory T cell subsets (reported above). It is perhaps not surprising that the CXCR4-using HXB2 infected a proportion of TSCM, as this virus can infect other memory CD4^+^ T cell subsets.

Investigation into the proportion of infected CD45RO^−^CD27^+^CCR7^+^ T cells which are TSCM (CD45RO^−^CD27^+^CCR7^+^CD95^+^CD122^+^) revealed that the majority of CD45RO^−^CD27^+^CCR7^+^ cells infected by CCR5-using JR-CSF were TSCM (infected mean CD45RO^−^CD27^+^CCR7^+^ 12.00 +/− 5.52%, TSCM 7.80 +/− 5.07%, 62 +/− 16% of CD45RO^−^CD27^+^CCR7^+^ T cells are TSCM, Figure 5B). In contrast, the minority of CD45RO^−^CD27^+^CCR7^+^ T cells infected by CXCR4-using HXB2 were TSCM (infected mean CD45RO^−^CD27^+^CCR7^+^ 5.13 +/− 2.00%, TSCM 6.32 +/− 1.65%, 25 +/− 7% of CD45RO^−^CD27^+^CCR7^+^ T cells are TSCM, Figure 5B). The overall percentages of TSCM infected by JR-CSF and HXB2 were similar; suggesting that it is the number of naïve T cells infected which increases with CXCR4-using viruses compared to CCR5-using viruses.

Infection of TSCM by CCR5- and CXCR4-using viruses *in vitro* has not been previously reported. This finding, combined with the reported potential of TSCM to be a long-lived reservoir for HIV-1 [12,16], is potentially important for the development of therapeutics targeting the HIV-1 reservoir for both CCR5- and CXCR4-using viruses. It is also important knowledge for therapies targeting subtype B HIV-1 strains, as approximately 40%–50% of subtype B viruses undergo a co-receptor switch during progression to advanced stages of infection [30,31], suggesting infection of TSCM could potentially be maintained throughout HIV-1 disease progression.

### 2.3. Measurement of Infection in CD4^+^ T Cell Subsets by HIV-1 Subtype C Viruses

We next tested the assay system using GFP reporter viruses pseudotyped with CCR5-using HIV-1 Envs isolated from two subjects infected with HIV-1 subtype C (subjects 258 and 1136). These Envs, and the clinical characteristics of the subjects have been described in detail recently [32,33]. The Env clones from subject 258 used here were 258-E-5, 258-E-6, 258-E-20 and 258-E-23, and those from subject 1136 were 1136-E-1, 1136-E-4, 1136-E-11 and 1136-E-12 [32,33]. All of the subtype C Envs exclusively used the CCR5 co-receptor [21,22].

**Figure 6:**
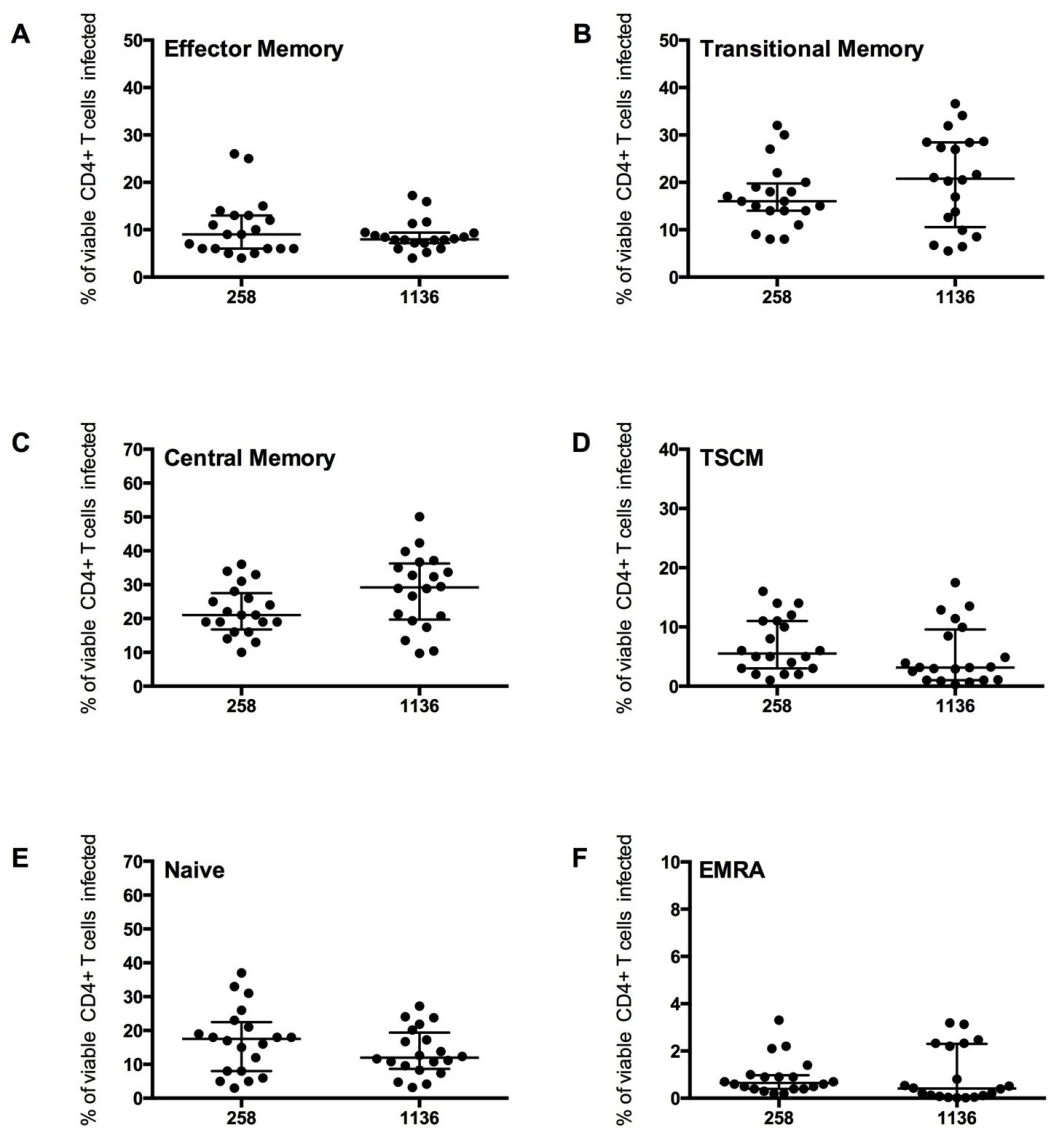
CD4^+^ T cell subset infection by HIV-1 subtype C Env pseudotyped GFP reporter viruses. CD4^+^ T cells from 5 healthy donors were infected with virus pseudotyped with 4 Envs from subject 258 and four from subject 1136, as described in the Experimental section. The percentage of infection for each CD4^+^ T cell subset was determined and quantified by flow cytometry (as shown in Figure 4). These CCR5-using viruses preferentially infected (**B**) TM and (**C**) CM T cells (mean level >17% infection), with a modest level of infection was seen in (**A**) EM (mean >8%), (**D**) TSCM (mean >5%) and (**E**) Naïve T cells (mean >13%). (**F**) EMRA were rarely infected (mean 1%). Scatter plots represent the median and interquartile range.

CD4^+^ T cells from five healthy donors were used to determine CD4^+^ T cell subset tropism of these viruses (Figure 6), using the strategy outlined in Figure 4. All HIV-1 subtype C Envs mediated comparable levels of CD4^+^ T cell infection (mean 2.0 ^+^/_−_ 0.7%), similar to levels demonstrated for the HIV-1 subtype B CCR5-using JR-CSF control virus (1.8 ^+^/_−_ 0.8%). All subtype C viruses preferentially infected memory T cell subsets, similar to JR-CSF; CM T cells were preferentially infected (mean 258 Envs 22.23 ^+^/_−_ 7.26%, 1136 Envs 28.30 ^+^/_−_ 10.89%), then TM (mean 258 Envs 17.10 ^+^/_−_ 6.59%, 1136 Envs 20.32 ^+^/_−_ 9.89%) and EM (mean 258 Envs 10.26 ^+^/_−_ 6.18%, 1136 Envs 8.79 ^+^/_−_ 3.22%, Figure 6A–C). Viruses from both subjects 258 and 1136 infected a similar proportion of TSCM (7 and 5% respectively), naïve T cells (16 and 13% respectively) and a lower proportion of EMRA cells (1%, Figure 6D–F).

Infection of the CD4^+^ T cell subsets by the subtype C viruses was similar to the trends shown by JR-CSF, with the highest proportion of cells infected being CM and TM. A similar level of EM, naïve and EMRA T cells were infected. The proportion of CD45RO^−^CD27^+^CCR7^+^ T cells infected which were TSCM cells was lower than the proportion infected by JR-CSF (mean JR-CSF 62 ^+^/_−_ 16.65%, 258 Envs 43.53 ^+^/_−_ 18.40%, 1136 Envs 32.97 ^+^/_−_ 30.94%). This finding could be influenced by a greater variability in TSCM cells infected by the different subtype C viruses (258 Envs 7.02 ^+^/_−_ 4.70%, 1136 Envs 5.28 ^+^/_−_ 5.12%), and perhaps across different subtypes of HIV-1 (JR-CSF mean 7.80 ^+^/_−_ 5.07%). More research into infection of TSCM by different HIV-1 subtypes is required to determine the factors influencing infection of this T cell subset.

## 3. Experimental Section

### 3.1. Cells

293T cells, JC53 cells [34] and TZM-bl cells [35] were maintained as described previously [21]. Peripheral blood mononuclear cells (PBMC) were purified from the blood of healthy HIV-1 negative donors by density gradient centrifugation.

### 3.2. HIV-1 Env Clones

All Envs used in this study are expressed from the pSVIII-Env mammalian expression plasmid [36]. For assay validation and as controls for each assay, the well characterized CCR5-using JR-CSF Env and CXCR4-using HXB2 Env were used, as described previously [21,25,26]. The HIV-1 subtype C Envs are derived from plasma of two subjects (subjects 258 and 1136) with chronic subtype C infection [33]. Four independent Envs from each subject were used in this study. The Env clones from subject 258 were 258-E-5, 258-E-6, 258-E-20 and 258-E-23, and those from subject 1136 were 1136-E-1, 1136-E-4, 1136-E-11 and 1136-E-12 [32,33]. These Envs were shown to be specific for the CCR5 co-receptor by phenotypic entry assays [33] and also by the recently developed CoRSeq_V3-C_ co-receptor usage prediction algorithm, that was designed specifically for HIV-1 subtype C Envs [37].

### 3.3. Production and Quantitation of Env Pseudotyped GFP Reporter Viruses

Env pseudotyped, GFP reporter viruses were produced as described previously [21]. Briefly, 293T cells were transfected with pNL4-3Env-GFP [38] and pSVIII-Env plasmids using Lipofectamine 2000 (Invitrogen, Carlsbad, CA, USA) at a ratio of 4:1. Supernatants were harvested 48 h later and filtered through 0.45 μm filters. Viruses were concentrated through a 20% (vol/vol) sucrose cushion, and stored at −80 °C. The TCID_50_ of virus stocks was determined by titration in TZM-bl cells, as described previously [35,39].

### 3.4. Enumeration of HIV-1 Infection in CD4^+^ T Cell Subsets

Forty-eight well tissue culture plates were seeded with 500 μL of 4 × 10^6^/mL of purified CD4^+^ T cells (2 × 10^6^ cells in each well) that were isolated from healthy donors using a RosetteSepCD4^+^ T-cell kit [Stemcell Technologies (Vancouver, BC, Canada), >95% purity of CD3^+^CD4^+^ T cells in each experiment]. Cells were suspended in RPMI 1640 medium containing 10% (vol/vol) FCS at all stages of the experiment. CD4^+^ T cells were incubated for 1 hour prior to infection with 3,000 infectious units of CCR5-using Env pseudotyped GFP reporter virus, or 1,250 infectious units of CXCR4-using virus by spinoculation (1,200 × *g* for 2 h) in V-bottom 96-well tissue culture plates. We empirically determined that this virus inoculum was within the linear range of infection for the CCR5- and CXCR4-using viruses used (data not shown).

Cells were then transferred to 48-well tissue culture plates and incubated for 3 days at 37 °C prior to staining with flow cytometry antibodies (Table 1). Flow cytometry antibodies were obtained from BD Biosciences (San Jose, CA, USA) with the exception of CD45RO eFlour450, CD122 PerCP-eFlour710 and the fixable viability dye eFlour506 which were from eBiosciences (San Diego, CA, USA). Cells were fixed for three hours in 4% (wt/vol) paraformaldehyde, then washed and resuspended in FACS buffer [filtered PBS with 2 mM EDTA and 0.5% (wt/vol) BSA]. We washed and suspended the cells in FACS buffer, as paraformaldehyde can cause changes in the emission of some fluorochromes, particularly APC-Cy7 dyes [40]. OneComp ebeads were used with the flow cytometry antibodies as compensation controls (eBiosciences).

HIV-1 infection was determined by CD3/GFP positivity and used to determine the distribution of infection within CD4^+^ T cell subsets, which were defined as naïve (CD45RO^−^CCR7^+^CD27^+^), TSCM (CD45RO^−^CCR7^+^CD27^+^CD95^+^CD122^+^), effector memory RA (EMRA, CD45RO^−^CCR7^−^CD27^−^), central memory (CM, CD45RO^+^CCR7^+^CD27^+^) effector memory (EM, CD45RO^+^CCR7^−^CD27^−^) and transitional memory (TM, CD45RO^+^CCR7^−^CD27^+^) T cells. This gating strategy allowed EM to be distinguished from TM [8,21] and superior enumeration of T cell subsets, as described previously [23,24]. TSCM were identified using methods similar to those described recently [11].

For these analyses, >1,000,000 events were collected on a LSR Fortessa flow cytometer (BD Biosciences) and analyzed with Flowlogic software (eBiosciences). The strategy for measuring HIV-1 infection in CD4^+^ T cell subsets is shown in Figure 1 and Figure 4, which are representative experiments of uninfected CD4^+^ T cells (Figure 1) and CD4^+^ T cells infected with GFP reporter virus pseudotyped with 258-E-5 Env (Figure 4), respectively.

## 4. Conclusions

We developed a novel *in vitro* CD4^+^ T cell infection assay to quantify the level and distribution of HIV-1 infection in CD4^+^ T cell subsets including the newly described TSCM subset. This assay was validated with CCR5-using and CXCR4-using viruses, and was able to distinguish distinct patterns of CD4^+^ T cell tropism associated with different co-receptor specificities. We further show that our assay can be used to measure CD4^+^ T cell subset infection by clinical isolates, specifically HIV-1 subtype C strains.

Our assay permits the simultaneous detection and quantification of HIV-1 infection in naïve, EMRA, TSCM, CM, TM, EM CD4^+^ T cell subsets. Investigation of changes in CD4^+^ T cell tropism by viruses isolated from longitudinal cohorts could potentially predict the establishment of viral reservoirs *in vivo*, and changes in cellular tropism that may be important for HIV-1 pathogenesis.
